# 979. Eligibility for and Use of Oral Antibiotic Therapy among Veterans with Osteo-articular Infections: A Retrospective Study across Eight Medical Centers

**DOI:** 10.1093/ofid/ofac492.821

**Published:** 2022-12-15

**Authors:** Jacquelyn Mareau, Bruce Alexander, Jason Egge, Brett Heintz, Hiroyuki Suzuki, Daniel J Livorsi

**Affiliations:** Iowa City VA Medical Center, Iowa City, Iowa; Iowa City VA Medical Center, Iowa City, Iowa; Iowa City VA Medical Center, Iowa City, Iowa; Iowa City VA Medical Center, Iowa City, Iowa; University of Iowa Carver College of Medicine, Iowa City, Iowa; University of Iowa Carver College of Medicine, Iowa City, Iowa

## Abstract

**Background:**

Osteoarticular infections are commonly treated with intravenous antibiotics even though oral antibiotics may be as effective. In this multicenter study, we evaluated how often patients with osteoarticular infections qualified for oral antibiotic therapy and how often oral antibiotics were prescribed.

**Methods:**

We randomly selected 2 high-complexity Veterans Affairs (VA) hospitals from each of the 4 US Census regions. Next, we randomly selected patients admitted to these 8 facilities during 2018-2020 who had a discharge diagnosis of an osteoarticular infection, received ≥5 days of inpatient antibiotics, and had an Infectious Disease consult. Manual chart reviews were performed to confirm the diagnosis and to exclude patients who received < 4 weeks of total antibiotics or had infection of the skull, sacrum or pelvis. Eligibility for oral therapy was defined by the inclusion criteria used in a recent randomized controlled non-inferiority trial. Oral antibiotic stepdown was defined as starting an exclusively oral regimen within 2 weeks of antibiotic initiation. The chi-square test was used to compare categorical variables.

**Results:**

After excluding 76 cases, the final cohort included 145 patients (median age 65, 98% male, 72% white). The most common types of osteo-articular infections were peripheral osteomyelitis (66%) and septic arthritis (14%). The most common isolated pathogen was *Staphylococcus aureus* (53%). Surgical debridement was performed in 104 (72%) patients, and the median antibiotic duration was 47 days (IQR 43-72). One hundred eight (74%) patients were eligible for oral step-down, but only 18 (17%) of these patients received it (Figure 1). Fourteen (78%) patients treated with oral step-down were from the same hospital. Among patients eligible for oral stepdown therapy, peripheral osteomyelitis was present in 15 (83%) of those who received oral therapy versus 58 (64%) of those who were eligible but did not receive it (p=0.12).

**Figure:**
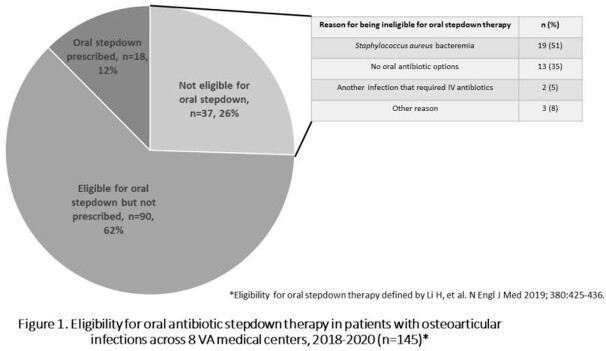

**Conclusion:**

In this retrospective study across 8 geographically-diverse VA hospitals, three-quarters of patients with osteoarticular infections qualified for oral antibiotics but only 1 of 6 eligible patients actually received them. Oral antibiotics may be under-utilized for treating osteoarticular infections in routine practice.

**Disclosures:**

**Daniel J. Livorsi, MD**, Merck & Co.: Grant/Research Support.

